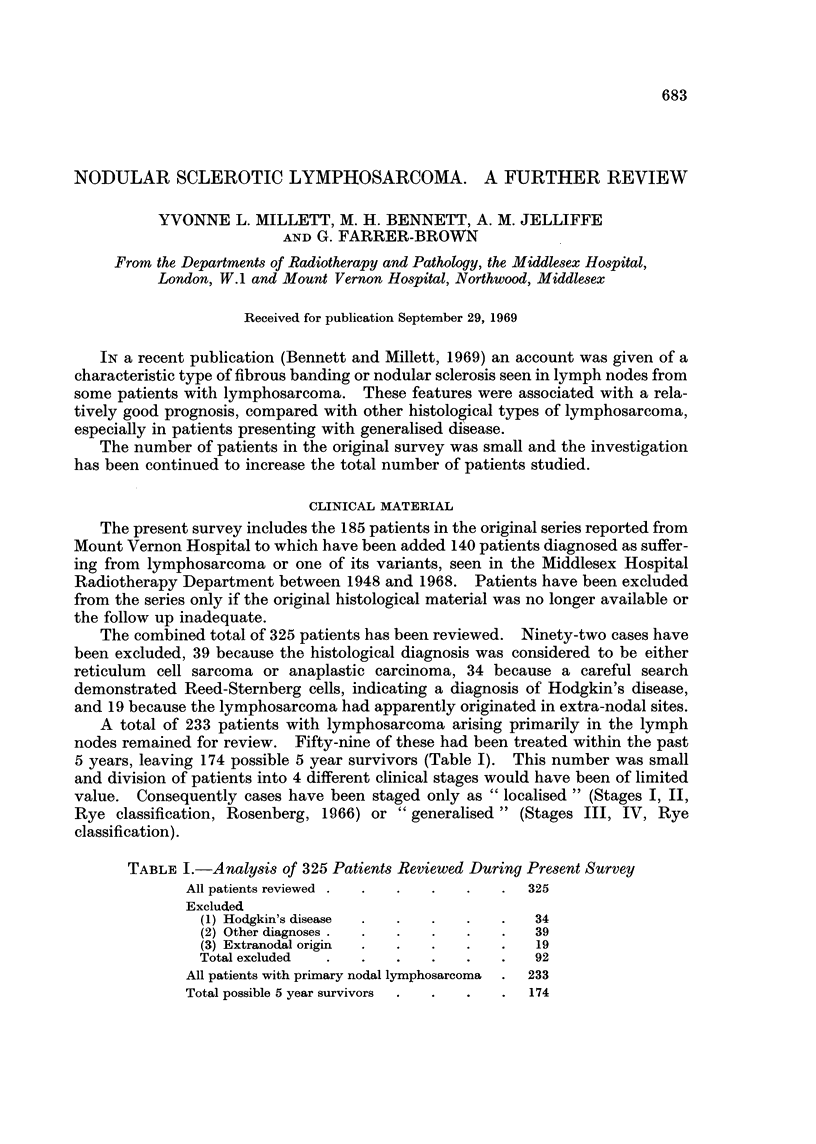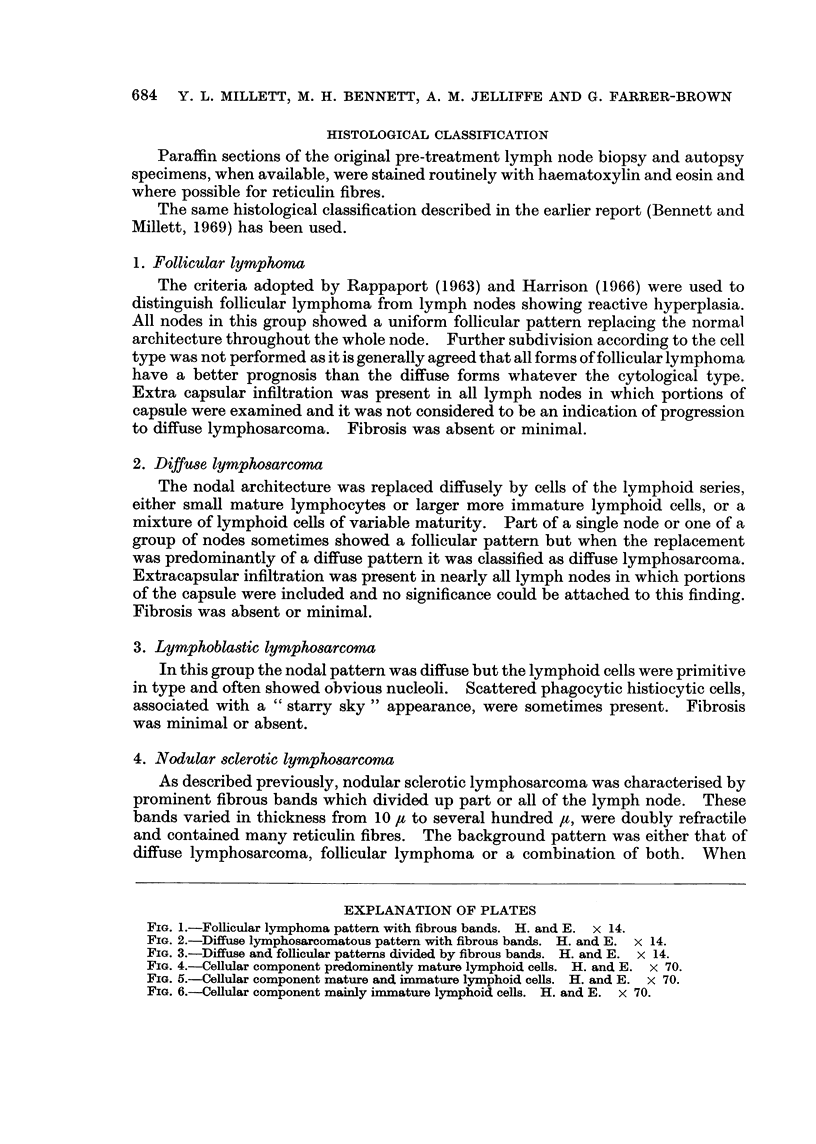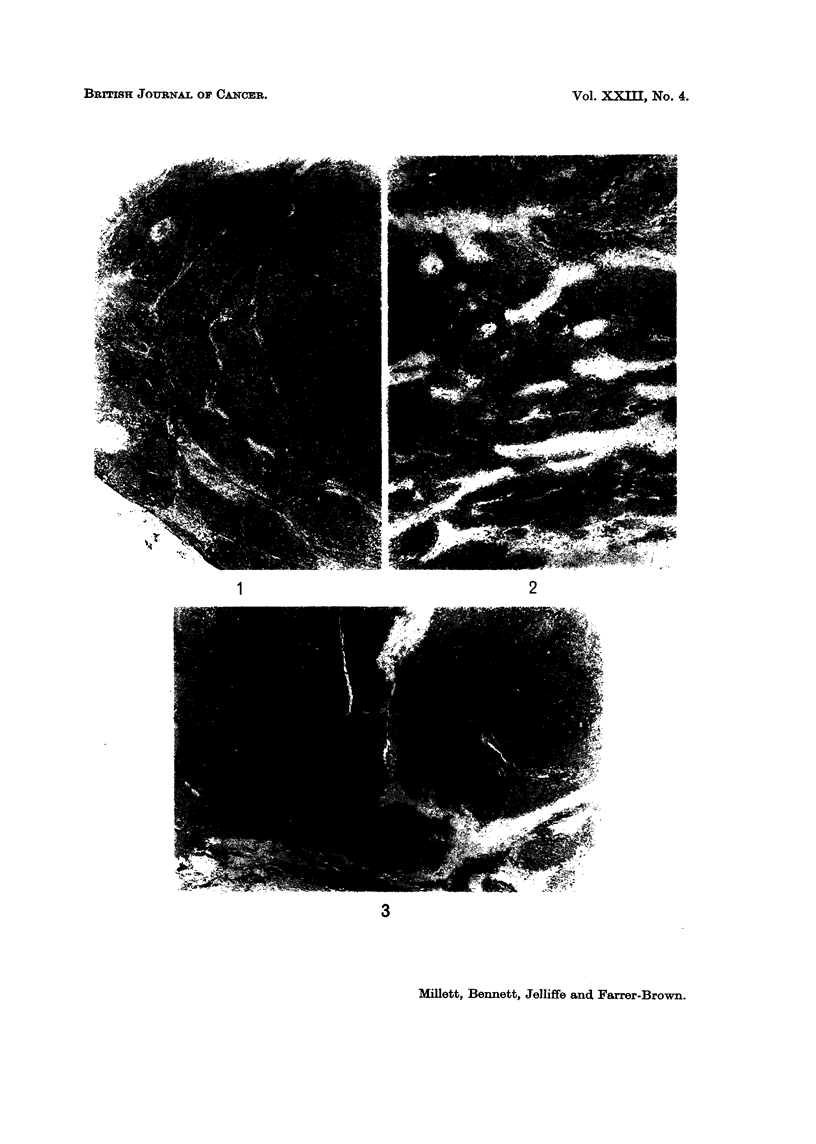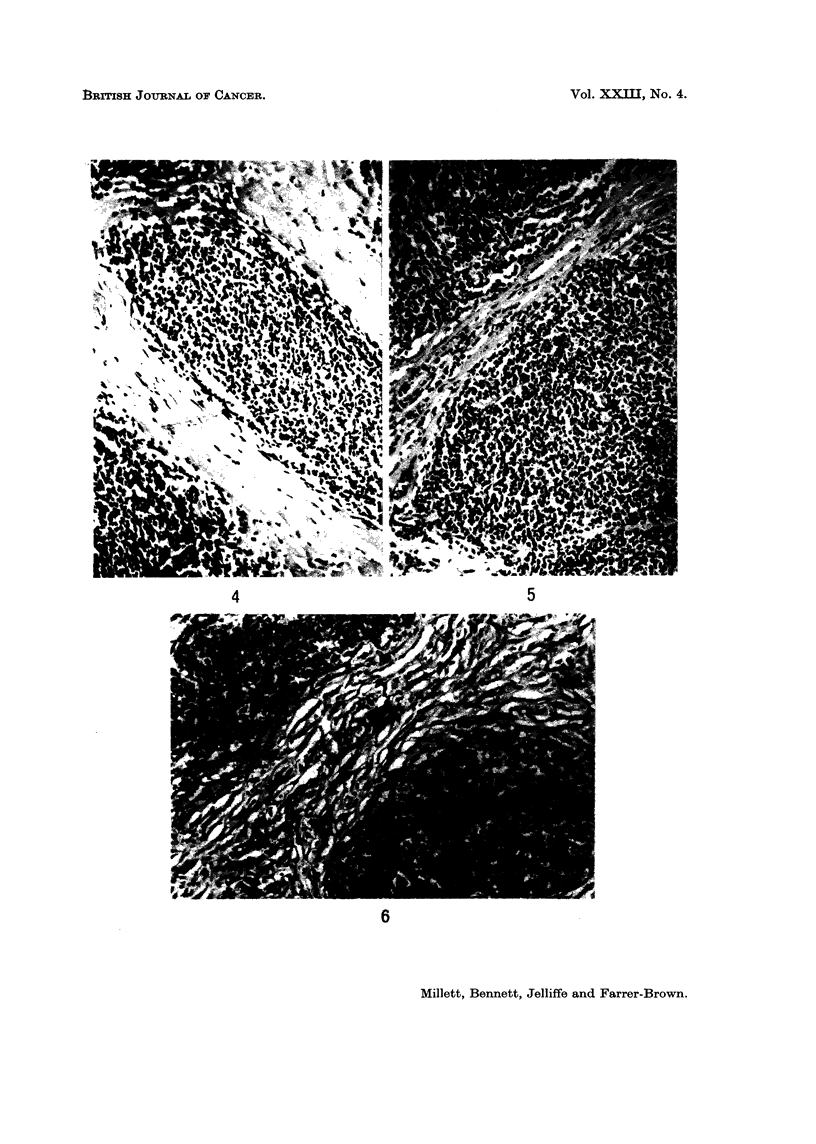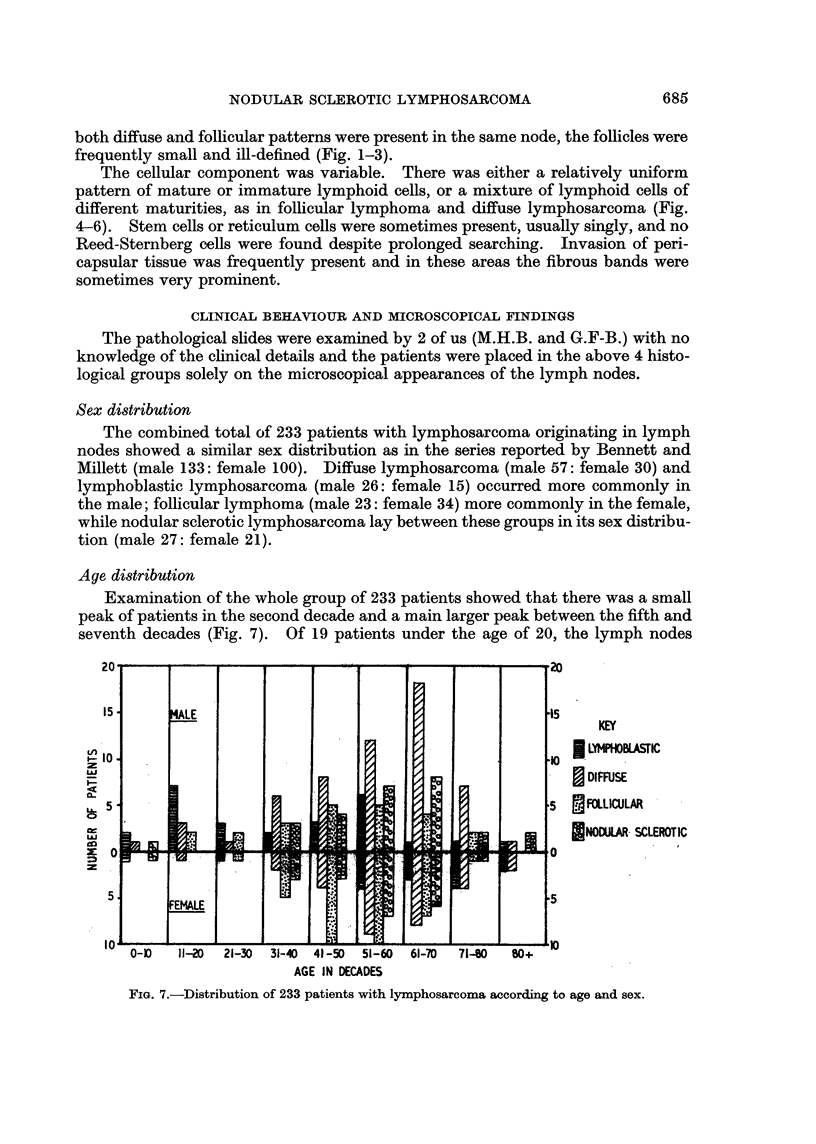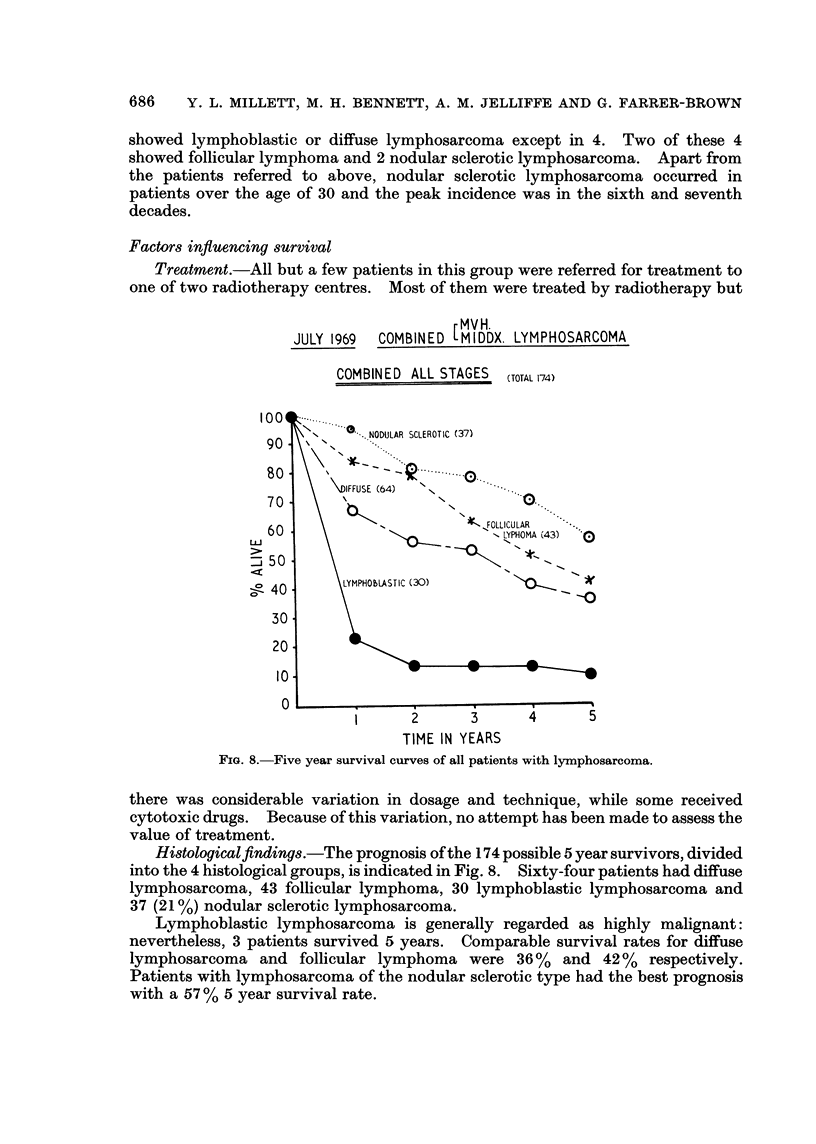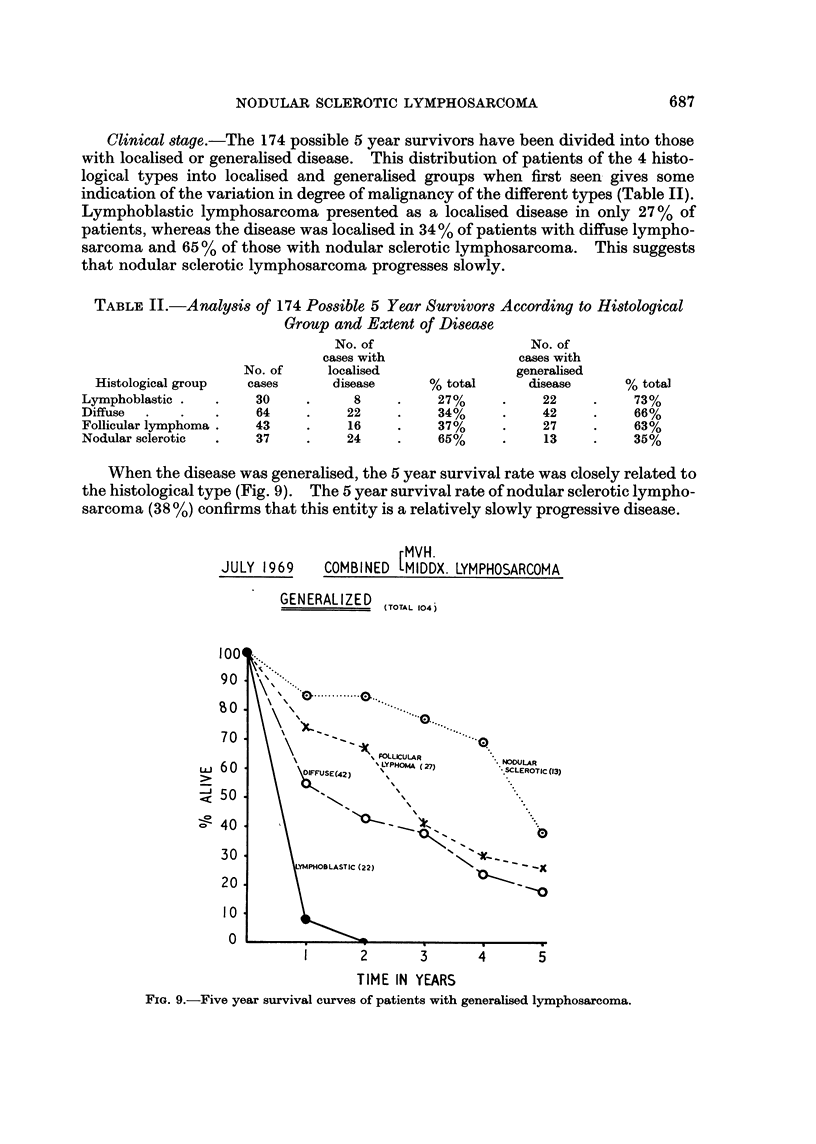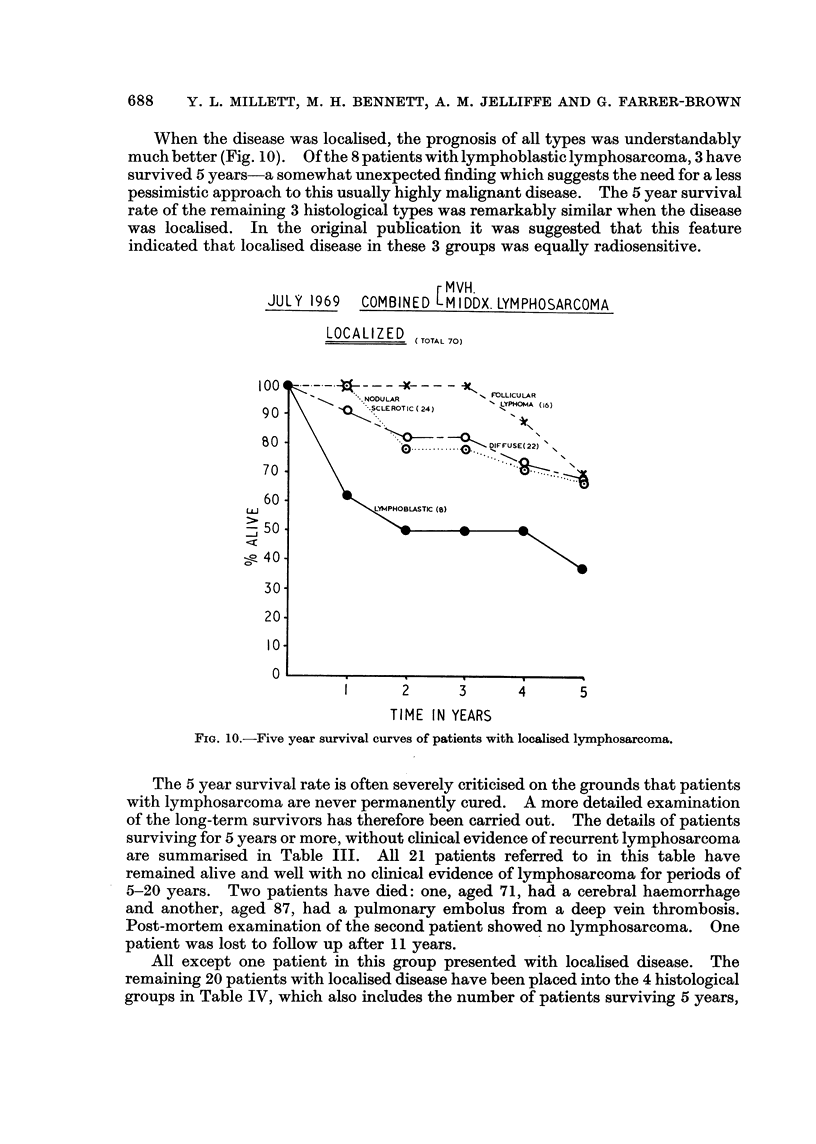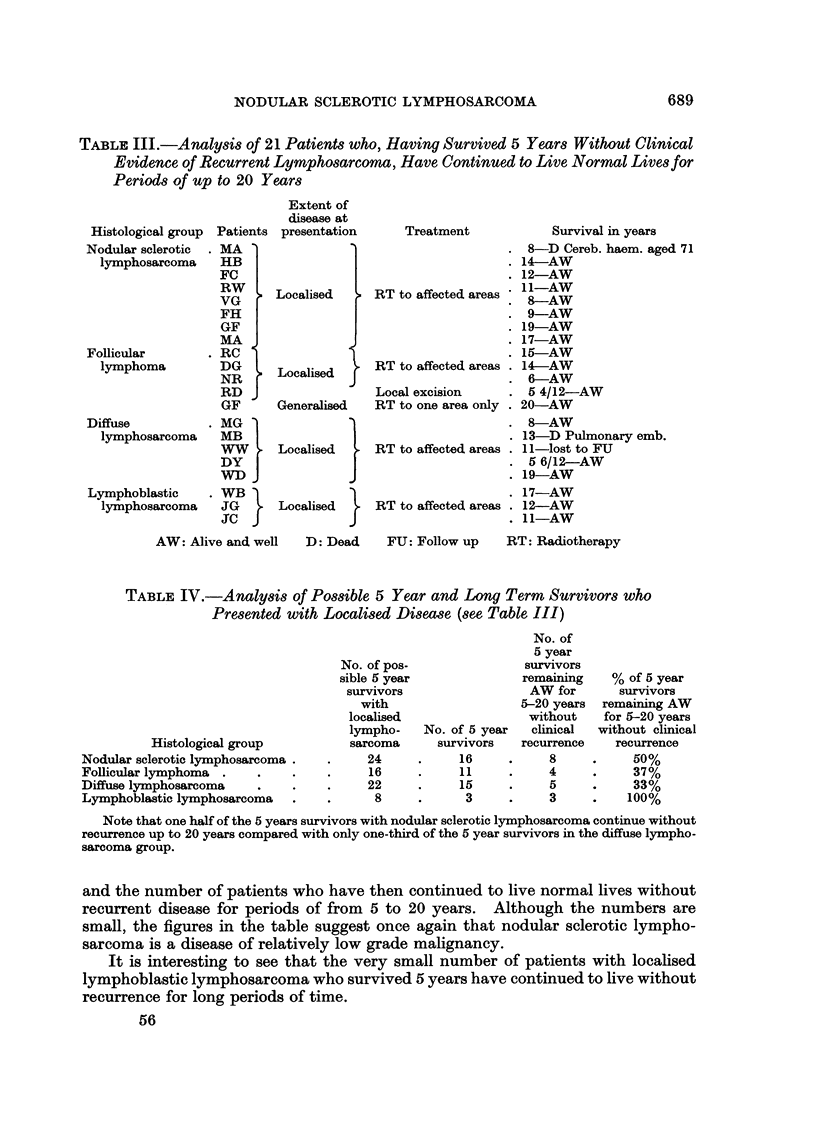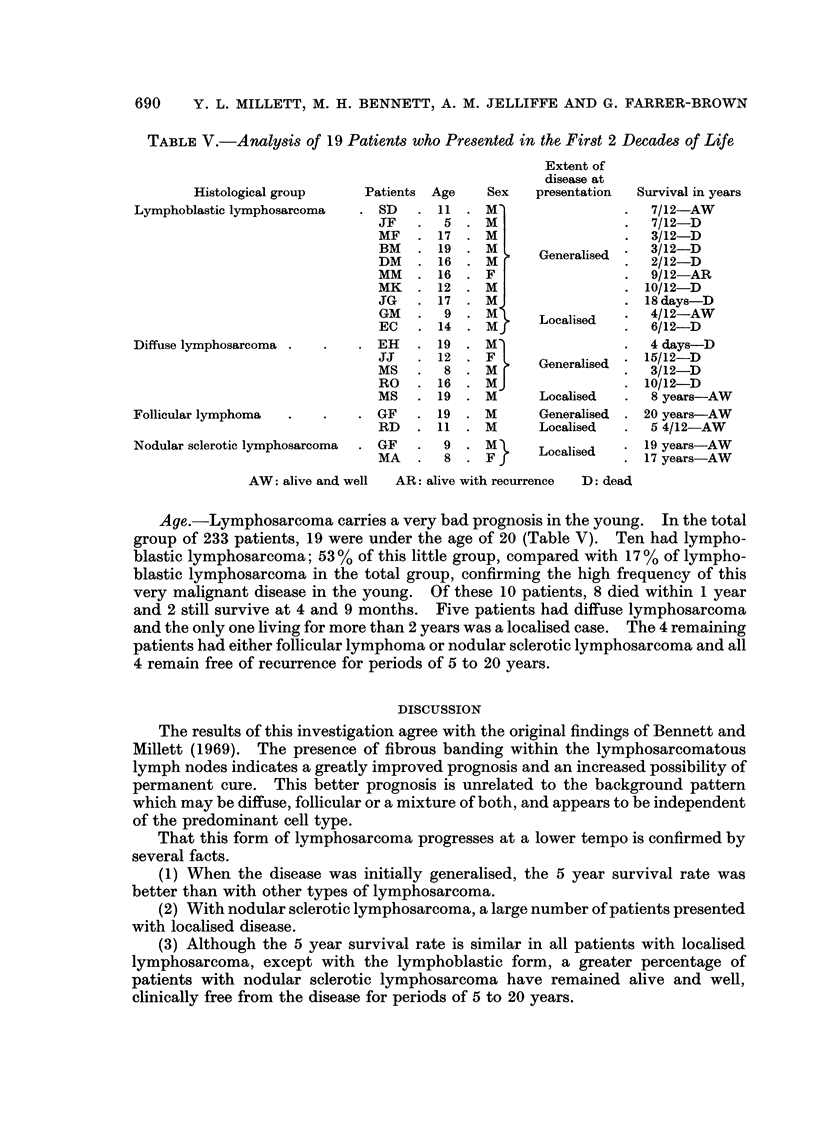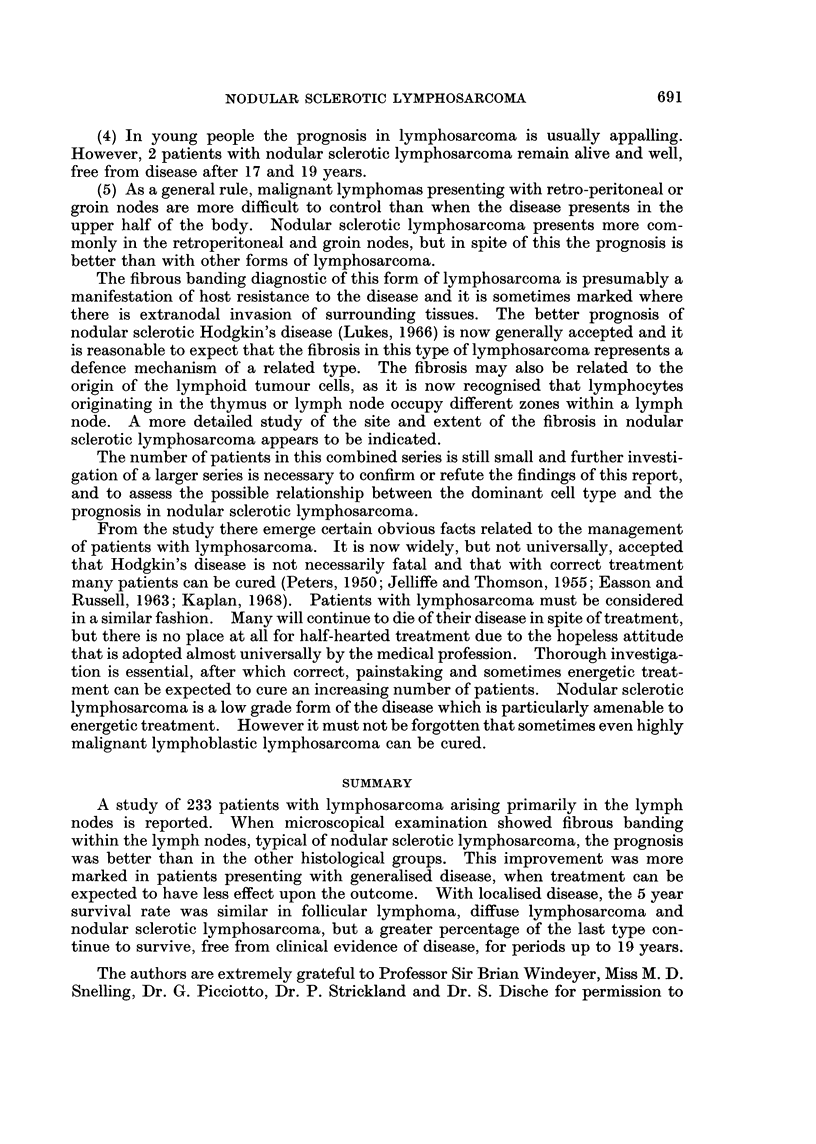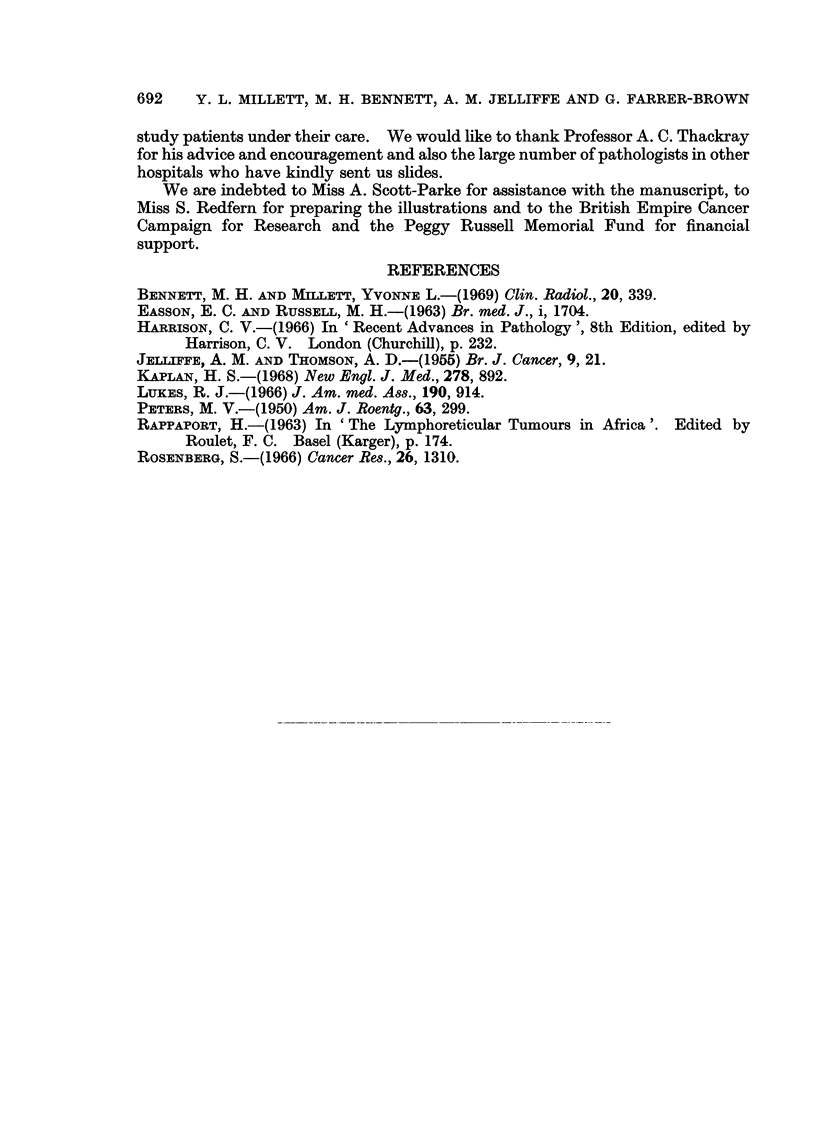# Nodular sclerotic lymphosarcoma. A further review.

**DOI:** 10.1038/bjc.1969.84

**Published:** 1969-12

**Authors:** Y. L. Millett, M. H. Bennett, A. M. Jelliffe, G. Farrer-Brown

## Abstract

**Images:**


### NODULAR SCLEROTIC LYMPHOSARCOMA. A FURTHER REVIEW

YVONNE L. MILLETT, M. H. BENNETT, A. M. JELLIFFE AND G. FARRER-BROWN

*From the Departments of Radiotherapy and Pathology, the Middlesex Hospital, London, W.1 and Mount Vernon Hospital, Northwood, Middlesex*

Received for publication September 29, 1969

IN a recent publication (Bennett and Millett, 1969) an account was given of a characteristic type of fibrous banding or nodular sclerosis seen in lymph nodes from some patients with lymphosarcoma. These features were associated with a relatively good prognosis, compared with other histological types of lymphosarcoma, especially in patients presenting with generalised disease.

The number of patients in the original survey was small and the investigation has been continued to increase the total number of patients studied.

### CLINICAL MATERIAL

The present survey includes the 185 patients in the original series reported from Mount Vernon Hospital to which have been added 140 patients diagnosed as suffering from lymphosarcoma or one of its variants, seen in the Middlesex Hospital Radiotherapy Department between 1948 and 1968. Patients have been excluded from the series only if the original histological material was no longer available or the follow up inadequate.

The combined total of 325 patients has been reviewed. Ninety-two cases have been excluded, 39 because the histological diagnosis was considered to be either reticulum cell sarcoma or anaplastic carcinoma, 34 because a careful search demonstrated Reed-Sternberg cells, indicating a diagnosis of Hodgkin's disease, and 19 because the lymphosarcoma had apparently originated in extra-nodal sites.

A total of 233 patients with lymphosarcoma arising primarily in the lymph nodes remained for review. Fifty-nine of these had been treated within the past 5 years, leaving 174 possible 5 year survivors (Table I). This number was small and division of patients into 4 different clinical stages would have been of limited value. Consequently cases have been staged only as " localised " (Stages I, II, Rye classification, Rosenberg, 1966) or " generalised " (Stages III, IV, Rye classification).

TABLE I.—*Analysis of* 325 *Patients Reviewed During Present Survey*

| | |
|---|---|
| All patients reviewed | 325 |
| Excluded | |
| (1) Hodgkin's disease | 34 |
| (2) Other diagnoses | 39 |
| (3) Extranodal origin | 19 |
| Total excluded | 92 |
| All patients with primary nodal lymphosarcoma | 233 |
| Total possible 5 year survivors | 174 |

### HISTOLOGICAL CLASSIFICATION

Paraffin sections of the original pre-treatment lymph node biopsy and autopsy specimens, when available, were stained routinely with haematoxylin and eosin and where possible for reticulin fibres.

The same histological classification described in the earlier report (Bennett and Millett, 1969) has been used.

### 1. *Follicular lymphoma*

The criteria adopted by Rappaport (1963) and Harrison (1966) were used to distinguish follicular lymphoma from lymph nodes showing reactive hyperplasia. All nodes in this group showed a uniform follicular pattern replacing the normal architecture throughout the whole node. Further subdivision according to the cell type was not performed as it is generally agreed that all forms of follicular lymphoma have a better prognosis than the diffuse forms whatever the cytological type. Extra capsular infiltration was present in all lymph nodes in which portions of capsule were examined and it was not considered to be an indication of progression to diffuse lymphosarcoma. Fibrosis was absent or minimal.

### 2. *Diffuse lymphosarcoma*

The nodal architecture was replaced diffusely by cells of the lymphoid series, either small mature lymphocytes or larger more immature lymphoid cells, or a mixture of lymphoid cells of variable maturity. Part of a single node or one of a group of nodes sometimes showed a follicular pattern but when the replacement was predominantly of a diffuse pattern it was classified as diffuse lymphosarcoma. Extracapsular infiltration was present in nearly all lymph nodes in which portions of the capsule were included and no significance could be attached to this finding. Fibrosis was absent or minimal.

### 3. *Lymphoblastic lymphosarcoma*

In this group the nodal pattern was diffuse but the lymphoid cells were primitive in type and often showed obvious nucleoli. Scattered phagocytic histiocytic cells, associated with a " starry sky " appearance, were sometimes present. Fibrosis was minimal or absent.

### 4. *Nodular sclerotic lymphosarcoma*

As described previously, nodular sclerotic lymphosarcoma was characterised by prominent fibrous bands which divided up part or all of the lymph node. These bands varied in thickness from 10 $\mu$ to several hundred $\mu$, were doubly refractile and contained many reticulin fibres. The background pattern was either that of diffuse lymphosarcoma, follicular lymphoma or a combination of both. When

---

EXPLANATION OF PLATES

FIG. 1.—Follicular lymphoma pattern with fibrous bands. H. and E. × 14.
FIG. 2.—Diffuse lymphosarcomatous pattern with fibrous bands. H. and E. × 14.
FIG. 3.—Diffuse and follicular patterns divided by fibrous bands. H. and E. × 14.
FIG. 4.—Cellular component predominently mature lymphoid cells. H. and E. × 70.
FIG. 5.—Cellular component mature and immature lymphoid cells. H. and E. × 70.
FIG. 6.—Cellular component mainly immature lymphoid cells. H. and E. × 70.

1

2

3

Millett, Bennett, Jelliffe and Farrer-Brown.

4

5

6

Millett, Bennett, Jelliffe and Farrer-Brown.

both diffuse and follicular patterns were present in the same node, the follicles were frequently small and ill-defined (Fig. 1–3).

The cellular component was variable. There was either a relatively uniform pattern of mature or immature lymphoid cells, or a mixture of lymphoid cells of different maturities, as in follicular lymphoma and diffuse lymphosarcoma (Fig. 4–6). Stem cells or reticulum cells were sometimes present, usually singly, and no Reed-Sternberg cells were found despite prolonged searching. Invasion of pericapsular tissue was frequently present and in these areas the fibrous bands were sometimes very prominent.

### CLINICAL BEHAVIOUR AND MICROSCOPICAL FINDINGS

The pathological slides were examined by 2 of us (M.H.B. and G.F-B.) with no knowledge of the clinical details and the patients were placed in the above 4 histological groups solely on the microscopical appearances of the lymph nodes.

### *Sex distribution*

The combined total of 233 patients with lymphosarcoma originating in lymph nodes showed a similar sex distribution as in the series reported by Bennett and Millett (male 133: female 100). Diffuse lymphosarcoma (male 57: female 30) and lymphoblastic lymphosarcoma (male 26: female 15) occurred more commonly in the male; follicular lymphoma (male 23: female 34) more commonly in the female, while nodular sclerotic lymphosarcoma lay between these groups in its sex distribution (male 27: female 21).

### *Age distribution*

Examination of the whole group of 233 patients showed that there was a small peak of patients in the second decade and a main larger peak between the fifth and seventh decades (Fig. 7). Of 19 patients under the age of 20, the lymph nodes

FIG. 7.—Distribution of 233 patients with lymphosarcoma according to age and sex.

showed lymphoblastic or diffuse lymphosarcoma except in 4. Two of these 4 showed follicular lymphoma and 2 nodular sclerotic lymphosarcoma. Apart from the patients referred to above, nodular sclerotic lymphosarcoma occurred in patients over the age of 30 and the peak incidence was in the sixth and seventh decades.

*Factors influencing survival*

*Treatment.*—All but a few patients in this group were referred for treatment to one of two radiotherapy centres. Most of them were treated by radiotherapy but

FIG. 8.—Five year survival curves of all patients with lymphosarcoma.

there was considerable variation in dosage and technique, while some received cytotoxic drugs. Because of this variation, no attempt has been made to assess the value of treatment.

*Histological findings.*—The prognosis of the 174 possible 5 year survivors, divided into the 4 histological groups, is indicated in Fig. 8. Sixty-four patients had diffuse lymphosarcoma, 43 follicular lymphoma, 30 lymphoblastic lymphosarcoma and 37 (21%) nodular sclerotic lymphosarcoma.

Lymphoblastic lymphosarcoma is generally regarded as highly malignant: nevertheless, 3 patients survived 5 years. Comparable survival rates for diffuse lymphosarcoma and follicular lymphoma were 36% and 42% respectively. Patients with lymphosarcoma of the nodular sclerotic type had the best prognosis with a 57% 5 year survival rate.

*Clinical stage.*—The 174 possible 5 year survivors have been divided into those with localised or generalised disease. This distribution of patients of the 4 histological types into localised and generalised groups when first seen gives some indication of the variation in degree of malignancy of the different types (Table II). Lymphoblastic lymphosarcoma presented as a localised disease in only 27% of patients, whereas the disease was localised in 34% of patients with diffuse lymphosarcoma and 65% of those with nodular sclerotic lymphosarcoma. This suggests that nodular sclerotic lymphosarcoma progresses slowly.

TABLE II.—*Analysis of* 174 *Possible* 5 *Year Survivors According to Histological Group and Extent of Disease*

| Histological group | No. of cases | No. of cases with localised disease | % total | No. of cases with generalised disease | % total |
|---|---|---|---|---|---|
| Lymphoblastic | 30 | 8 | 27% | 22 | 73% |
| Diffuse | 64 | 22 | 34% | 42 | 66% |
| Follicular lymphoma | 43 | 16 | 37% | 27 | 63% |
| Nodular sclerotic | 37 | 24 | 65% | 13 | 35% |

When the disease was generalised, the 5 year survival rate was closely related to the histological type (Fig. 9). The 5 year survival rate of nodular sclerotic lymphosarcoma (38%) confirms that this entity is a relatively slowly progressive disease.

FIG. 9.—Five year survival curves of patients with generalised lymphosarcoma.

When the disease was localised, the prognosis of all types was understandably much better (Fig. 10). Of the 8 patients with lymphoblastic lymphosarcoma, 3 have survived 5 years—a somewhat unexpected finding which suggests the need for a less pessimistic approach to this usually highly malignant disease. The 5 year survival rate of the remaining 3 histological types was remarkably similar when the disease was localised. In the original publication it was suggested that this feature indicated that localised disease in these 3 groups was equally radiosensitive.

FIG. 10.—Five year survival curves of patients with localised lymphosarcoma.

The 5 year survival rate is often severely criticised on the grounds that patients with lymphosarcoma are never permanently cured. A more detailed examination of the long-term survivors has therefore been carried out. The details of patients surviving for 5 years or more, without clinical evidence of recurrent lymphosarcoma are summarised in Table III. All 21 patients referred to in this table have remained alive and well with no clinical evidence of lymphosarcoma for periods of 5–20 years. Two patients have died: one, aged 71, had a cerebral haemorrhage and another, aged 87, had a pulmonary embolus from a deep vein thrombosis. Post-mortem examination of the second patient showed no lymphosarcoma. One patient was lost to follow up after 11 years.

All except one patient in this group presented with localised disease. The remaining 20 patients with localised disease have been placed into the 4 histological groups in Table IV, which also includes the number of patients surviving 5 years,

TABLE III.—*Analysis of 21 Patients who, Having Survived 5 Years Without Clinical Evidence of Recurrent Lymphosarcoma, Have Continued to Live Normal Lives for Periods of up to 20 Years*

| Histological group | Patients | Extent of disease at presentation | Treatment | Survival in years |
|---|---|---|---|---|
| Nodular sclerotic lymphosarcoma | MA | Localised | RT to affected areas | 8—D Cereb. haem. aged 71 |
| | HB | | | 14—AW |
| | FC | | | 12—AW |
| | RW | | | 11—AW |
| | VG | | | 8—AW |
| | FH | | | 9—AW |
| | GF | | | 19—AW |
| | MA | | | 17—AW |
| Follicular lymphoma | RC | Localised | RT to affected areas | 15—AW |
| | DG | | | 14—AW |
| | NR | | | 6—AW |
| | RD | | Local excision | 5 4/12—AW |
| | GF | Generalised | RT to one area only | 20—AW |
| Diffuse lymphosarcoma | MG | Localised | RT to affected areas | 8—AW |
| | MB | | | 13—D Pulmonary emb. |
| | WW | | | 11—lost to FU |
| | DY | | | 5 6/12—AW |
| | WD | | | 19—AW |
| Lymphoblastic lymphosarcoma | WB | Localised | RT to affected areas | 17—AW |
| | JG | | | 12—AW |
| | JC | | | 11—AW |

AW: Alive and well D: Dead FU: Follow up RT: Radiotherapy

TABLE IV.—*Analysis of Possible 5 Year and Long Term Survivors who Presented with Localised Disease (see Table III)*

| Histological group | No. of possible 5 year survivors with localised lymphosarcoma | No. of 5 year survivors | No. of 5 year survivors remaining AW for 5–20 years without clinical recurrence | % of 5 year survivors remaining AW for 5–20 years without clinical recurrence |
|---|---|---|---|---|
| Nodular sclerotic lymphosarcoma | 24 | 16 | 8 | 50% |
| Follicular lymphoma | 16 | 11 | 4 | 37% |
| Diffuse lymphosarcoma | 22 | 15 | 5 | 33% |
| Lymphoblastic lymphosarcoma | 8 | 3 | 3 | 100% |

Note that one half of the 5 years survivors with nodular sclerotic lymphosarcoma continue without recurrence up to 20 years compared with only one-third of the 5 year survivors in the diffuse lymphosarcoma group.

and the number of patients who have then continued to live normal lives without recurrent disease for periods of from 5 to 20 years. Although the numbers are small, the figures in the table suggest once again that nodular sclerotic lymphosarcoma is a disease of relatively low grade malignancy.

It is interesting to see that the very small number of patients with localised lymphoblastic lymphosarcoma who survived 5 years have continued to live without recurrence for long periods of time.

TABLE V.—*Analysis of* 19 *Patients who Presented in the First* 2 *Decades of Life*

| Histological group | Patients | Age | Sex | Extent of disease at presentation | Survival in years |
|---|---|---|---|---|---|
| Lymphoblastic lymphosarcoma | SD | 11 | M | Generalised | 7/12—AW |
| | JF | 5 | M | | 7/12—D |
| | MF | 17 | M | | 3/12—D |
| | BM | 19 | M | | 3/12—D |
| | DM | 16 | M | | 2/12—D |
| | MM | 16 | F | | 9/12—AR |
| | MK | 12 | M | | 10/12—D |
| | JG | 17 | M | | 18 days—D |
| | GM | 9 | M | Localised | 4/12—AW |
| | EC | 14 | M | | 6/12—D |
| Diffuse lymphosarcoma | EH | 19 | M | Generalised | 4 days—D |
| | JJ | 12 | F | | 15/12—D |
| | MS | 8 | M | | 3/12—D |
| | RO | 16 | M | | 10/12—D |
| | MS | 19 | M | Localised | 8 years—AW |
| Follicular lymphoma | GF | 19 | M | Generalised | 20 years—AW |
| | RD | 11 | M | Localised | 5 4/12—AW |
| Nodular sclerotic lymphosarcoma | GF | 9 | M | Localised | 19 years—AW |
| | MA | 8 | F | | 17 years—AW |

AW: alive and well AR: alive with recurrence D: dead

*Age.*—Lymphosarcoma carries a very bad prognosis in the young. In the total group of 233 patients, 19 were under the age of 20 (Table V). Ten had lymphoblastic lymphosarcoma; 53% of this little group, compared with 17% of lymphoblastic lymphosarcoma in the total group, confirming the high frequency of this very malignant disease in the young. Of these 10 patients, 8 died within 1 year and 2 still survive at 4 and 9 months. Five patients had diffuse lymphosarcoma and the only one living for more than 2 years was a localised case. The 4 remaining patients had either follicular lymphoma or nodular sclerotic lymphosarcoma and all 4 remain free of recurrence for periods of 5 to 20 years.

### DISCUSSION

The results of this investigation agree with the original findings of Bennett and Millett (1969). The presence of fibrous banding within the lymphosarcomatous lymph nodes indicates a greatly improved prognosis and an increased possibility of permanent cure. This better prognosis is unrelated to the background pattern which may be diffuse, follicular or a mixture of both, and appears to be independent of the predominant cell type.

That this form of lymphosarcoma progresses at a lower tempo is confirmed by several facts.

(1) When the disease was initially generalised, the 5 year survival rate was better than with other types of lymphosarcoma.

(2) With nodular sclerotic lymphosarcoma, a large number of patients presented with localised disease.

(3) Although the 5 year survival rate is similar in all patients with localised lymphosarcoma, except with the lymphoblastic form, a greater percentage of patients with nodular sclerotic lymphosarcoma have remained alive and well, clinically free from the disease for periods of 5 to 20 years.

(4) In young people the prognosis in lymphosarcoma is usually appalling. However, 2 patients with nodular sclerotic lymphosarcoma remain alive and well, free from disease after 17 and 19 years.

(5) As a general rule, malignant lymphomas presenting with retro-peritoneal or groin nodes are more difficult to control than when the disease presents in the upper half of the body. Nodular sclerotic lymphosarcoma presents more commonly in the retroperitoneal and groin nodes, but in spite of this the prognosis is better than with other forms of lymphosarcoma.

The fibrous banding diagnostic of this form of lymphosarcoma is presumably a manifestation of host resistance to the disease and it is sometimes marked where there is extranodal invasion of surrounding tissues. The better prognosis of nodular sclerotic Hodgkin's disease (Lukes, 1966) is now generally accepted and it is reasonable to expect that the fibrosis in this type of lymphosarcoma represents a defence mechanism of a related type. The fibrosis may also be related to the origin of the lymphoid tumour cells, as it is now recognised that lymphocytes originating in the thymus or lymph node occupy different zones within a lymph node. A more detailed study of the site and extent of the fibrosis in nodular sclerotic lymphosarcoma appears to be indicated.

The number of patients in this combined series is still small and further investigation of a larger series is necessary to confirm or refute the findings of this report, and to assess the possible relationship between the dominant cell type and the prognosis in nodular sclerotic lymphosarcoma.

From the study there emerge certain obvious facts related to the management of patients with lymphosarcoma. It is now widely, but not universally, accepted that Hodgkin's disease is not necessarily fatal and that with correct treatment many patients can be cured (Peters, 1950; Jelliffe and Thomson, 1955; Easson and Russell, 1963; Kaplan, 1968). Patients with lymphosarcoma must be considered in a similar fashion. Many will continue to die of their disease in spite of treatment, but there is no place at all for half-hearted treatment due to the hopeless attitude that is adopted almost universally by the medical profession. Thorough investigation is essential, after which correct, painstaking and sometimes energetic treatment can be expected to cure an increasing number of patients. Nodular sclerotic lymphosarcoma is a low grade form of the disease which is particularly amenable to energetic treatment. However it must not be forgotten that sometimes even highly malignant lymphoblastic lymphosarcoma can be cured.

### SUMMARY

A study of 233 patients with lymphosarcoma arising primarily in the lymph nodes is reported. When microscopical examination showed fibrous banding within the lymph nodes, typical of nodular sclerotic lymphosarcoma, the prognosis was better than in the other histological groups. This improvement was more marked in patients presenting with generalised disease, when treatment can be expected to have less effect upon the outcome. With localised disease, the 5 year survival rate was similar in follicular lymphoma, diffuse lymphosarcoma and nodular sclerotic lymphosarcoma, but a greater percentage of the last type continue to survive, free from clinical evidence of disease, for periods up to 19 years.

The authors are extremely grateful to Professor Sir Brian Windeyer, Miss M. D. Snelling, Dr. G. Picciotto, Dr. P. Strickland and Dr. S. Dische for permission to study patients under their care. We would like to thank Professor A. C. Thackray for his advice and encouragement and also the large number of pathologists in other hospitals who have kindly sent us slides.

We are indebted to Miss A. Scott-Parke for assistance with the manuscript, to Miss S. Redfern for preparing the illustrations and to the British Empire Cancer Campaign for Research and the Peggy Russell Memorial Fund for financial support.

### REFERENCES

BENNETT, M. H. AND MILLETT, YVONNE L.—(1969) *Clin. Radiol.*, **20**, 339.
EASSON, E. C. AND RUSSELL, M. H.—(1963) *Br. med. J.*, i, 1704.
HARRISON, C. V.—(1966) In ' Recent Advances in Pathology ', 8th Edition, edited by Harrison, C. V. London (Churchill), p. 232.
JELLIFFE, A. M. AND THOMSON, A. D.—(1955) *Br. J. Cancer*, **9**, 21.
KAPLAN, H. S.—(1968) *New Engl. J. Med.*, **278**, 892.
LUKES, R. J.—(1966) *J. Am. med. Ass.*, **190**, 914.
PETERS, M. V.—(1950) *Am. J. Roentg.*, **63**, 299.
RAPPAPORT, H.—(1963) In ' The Lymphoreticular Tumours in Africa '. Edited by Roulet, F. C. Basel (Karger), p. 174.
ROSENBERG, S.—(1966) *Cancer Res.*, **26**, 1310.